# Novel bi-allelic DNAH3 variants cause oligoasthenoteratozoospermia

**DOI:** 10.3389/fendo.2024.1462509

**Published:** 2024-10-28

**Authors:** Shu Li, Zexin Zhang, Linna Xie, Yanqiu Zhao, Hongtai Chen, Shijia Zhang, Yixiang Cai, Bingjie Ren, Wensheng Liu, Songxi Tang, Yanwei Sha

**Affiliations:** ^1^ Department of Andrology, Women and Children’s Hospital, School of Medicine, Xiamen University, Xiamen, Fujian, China; ^2^ Peking Union Medical College, Chinese Academy of Medical Sciences, Beijing, China; ^3^ State Key Laboratory of Molecular Vaccinology and Molecular Diagnostics, School of Public Health, Xiamen University, Xiamen, Fujian, China; ^4^ National Health Commission (NHC) Key Laboratory of Male Reproduction and Genetics, Guangdong Provincial Reproductive Science Institute (Guangdong Provincial Fertility Hospital), Guangzhou, Guangdong, China; ^5^ Department of Andrology and Sexual Medicine, First Affiliated Hospital of Fujian Medical University, Fuzhou, China; ^6^ Fujian Provincial Key Laboratory of Reproductive Health Research, School of Medicine, Xiamen University, Xiamen, Fujian, China

**Keywords:** oligoasthenoteratozoospermia, dynein, intracytoplasmic sperm injection, male infertility, DNAH3

## Abstract

**Background:**

Oligoasthenoteratozoospermia (OAT) is a widespread cause of male infertility. One of the usual clinical manifestations of OAT is multiple morphological abnormalities of the sperm flagella (MMAF), which are frequently associated with mutations and defects in the dynein family. However, the relationship between the newly identified Dynein Axonemal Heavy Chain 3 (DNAH3) mutation and oligonasthenospermia in humans has not yet been established.

**Methods:**

Whole exome sequencing, pathogenicity analysis, and species conservation analysis of mutation sites were conducted on two patients from different unrelated families with DNAH3 mutations. We identified representative mutation sites and predicted the protein structure following these mutations. The sperm characteristics of the two patients with DNAH3 mutations were verified using Papanicolaou staining, scanning electron microscopy, and transmission electron microscopy. Additionally, mRNA and protein levels were assessed through RT-qPCR and Western blotting.

**Results:**

The biallelic mutations in the first progenitor included a heterozygous deletion and insertion, c.6535_6536 delinsAC (to infect mutation (p.Asp2179Thr), and stop codon premutation, c.3249G > A (p.Trp1083Ter). In Family II, the patient (P2) harbored a DNAH3 heterozygous missense mutation, c. 10439G> A(p.Arg3480Gln), along with a stop codon premutation, (c.10260G > A; p.Trp3420Ter). Patients with premature termination of transcription or translation due to DNAH3 mutations exhibit OAT phenotypes, including fibrous sheath dysplasia and multiple tail malformations. We identified the representative sites after mutation, predicted the protein structure, and assessed changes in the protein levels of DNAH3 and related genes following mutations. Notably,a significant reduction in DNAH3 protein expression was validated in these patients. We may explore in the future how DNAH3 affects sperm motility and quality through regulatory mechanisms involving protein structural changes.

**Conclusion:**

Novel biallelic mutations in DNAH3, especially those resulting in a premature stop codon, may alter protein expression, structure, and active site, leading to spermatogenic failure and potentially inducing OAT. The discovery of new mutations in DNAH3 may be the key to the diagnosis and treatment of OAT.

## 1 Introduction

Men with oligoasthenoteratozoospermia (OAT) account for 30% of the millions of couples who have difficulties in conceiving, making it a major world reproductive health problem (1, 2). The genetic basis of this condition in many affected individuals remains unknown. According to the indicators outlined in the sixth edition of the WHO Manual for Laboratory Examination of Human Sperm (3, 4), patients diagnosed with OAT often present with male infertility, and their spermatozoa are characterized by reduced sperm concentration, a decreased quantity of motile and progressively motile sperm, and multiple abnormalities in the sperm head, neck, and tail flagellum (5, 6). It has long been recognized that genes influencing spermatogenesis and sperm motility capacitation processes are crucial.

Currently, several genes associated with dynein family proteins (7–9), fibrous sheath proteins (10–12), and mitochondrial sheath-related genes (13) have been identified as being involved with these processes. In particular, the Dynein Axonemal Heavy Chain (DNAH) family is known to be essential for spermatogenesis (8, 14), structural integrity (15–19), flagellar formation (6, 20, 21), and motility (14, 22).

Spermatids begin to develop flagella, which are essential structures for sperm motility. The formation of flagella involves the precise assembly of microtubules and various proteins (including the DNAH protein family) to create functional motile organelles. Concurrently, the cytoplasm begins to reduce, and organelle are rearranged. During maturation, spermatids shed most of their cytoplasm, and the rearrangement of organelles supports the motility and fertilization functions of the sperm. Errors in these processes can lead to male infertility (23). Therefore, defects in Dynein Axonemal Heavy Chain 3 (DNAH3), a member of the axonal dynein family that plays an important role in the structure and beating frequency of flagella, may interfere with processes such as flagella formation and sperm nucleus assembly.

In this study, whole-exome sequencing (WES) revealed biallelic compound heterozygous mutations in DNAH3 in two unrelated Chinese male patients with OAT. Apart from these mutations, they did not carry any known biallelic pathogenic mutations. Mammalian DNAH3 is a member of the dynein axonemal heavy chain family, encoding a specific axonemal dynein involved in material transport and protein assembly in eukaryotic cells (24, 25). It is primarily expressed in the testis and is associated with sperm flagellar assembly and motility. Genes related to sperm flagellar assembly and motility, including the DNAH family genes and testis-specific candidate marker genes, have previously been shown to have lower expression levels in the testes of infertile triploid fish (26), suggesting a close relationship with male reproduction.

## 2 Materials and methods

### 2.1 Human subject collection and information

A total of 128 unrelated men with a preliminary diagnosis of OAT were recruited from the Women and Children’s Hospital of Xiamen University, China, from January 2023 to April 2024. Some of the patients in this cohort have been described in previous publications (27). All the men involved in the study had primary infertility for over 1 year, characterized by 1 year of normal sex without contraception and unsuccessful attempts to conceive naturally. Two patients in this study came from families with no close familial relationships, with ages of 29 years (P1) and 32 years (P2). Careful clinical examination showed no significant symptoms of other diseases related to ciliary dysfunction, such as primary ciliate dyskinesia or Bardet–Biedl syndrome, in either case. Both patients showed a normal male karyotype (46, XY). Informed consent was obtained from each subject.

Semen analysis is performed during routine examinations of individuals following WHO guidelines (Sixth Edition). Spermatozoa morphology was observed using modified Papanicolaou staining, with at least 200 to 300 sperm examined. The percentage of sperm with abnormal morphology was assessed according to WHO guidelines.

### 2.2 Whole-exome sequencing

As previously mentioned, whole exome sequencing (WES) was conducted on these OAT patients. Genomic DNA was isolated from the peripheral blood samples of each patient and sequenced using the Illumina Hiseq 2000 sequencer. The sequencing reads were aligned to the Human Genome Reference (hg19) using the Burrows–Wheeler Aligner and sorted with Picard software. Candidate variants were annotated using ANNOVAR and other bioinformatics databases. Sanger sequencing was performed to verify specific mutation sites in patients with DNAH3 mutations. We also verified the exon sequencing of the parents of two young and middle-aged patients.

### 2.3 Protein structure prediction

We used an online protein 3D structure prediction website (28–32) (https://swissmodel.expasy.org/) and employed homology modeling to analyze two DNAH3 variants with premature stop codons, comparing them with the wild-type protein variant. The structures were visualized with PyMOL 2.6 (33), and we selected the site with the highest comprehensive score among the predicted protein activity pockets in Protein Plus (34, 35) for display.

### 2.4 Electron microscopy analysis

Transmission electron microscopy (TEM) was used for sample processing and imaging at the biomedical instrument platform of Xiamen University. First, the sperm was washed and centrifuged, and the fresh, unfrozen sperm was fixed with 2.5% glutaraldehyde. The sample was washed three times with 0.1 M phosphate buffer and then suspended in 0.2 M sodium carboxylate buffer. After embedding in Epon 812, ultrathin sections were stained with uranyl acetate and lead citrate, and observed using transmission electron microscopy (JEM-1400, Hitachi High-Tech, Japan).

Sample treatment for scanning electron microscopy (SEM) involved smearing the washed sperm, fixing it with glutaraldehyde, and subjecting it to gradient dehydration. The samples were then dried and coated with metal before being observed using a focused ion beam scanning electron microscope (Helios 5 UC, ThermoFisher, USA). In this study, the neck, middle section, main segment, terminal segment, and fibrous sheath of sperm were observed using SEM and TEM. More than 200 sperm were observed and counted, focusing on local scanning phenotypes and longitudinal sections, confirming the phenotypes associated with MMAF.

### 2.5 Total RNA extraction and reverse-transcription PCR

Total RNA was extracted from mouse tissues using Trizol (Invitrogen, Carlsbad, USA). The extracted RNA was then reverse transcribed into cDNA using a One-Step Reverse Transcription Kit (ABM, Vancouver, Canada), followed by a 10-fold dilution. Subsequently, qPCR was conducted using SYBR qPCR Mix (Bio-Rad), with Gapdh was utilized as an internal control. The data were analyzed using the 2^−(ΔΔCt) method, and the specific primers are listed in **Supplementary Table S1**.

### 2.6 Western blotting

Patient semen, after centrifugation, and freshly extracted normal mouse testicles were homogenized using an ultrasound homogenizer with the addition of a moderate amount of 100× protease inhibitor (MCE, New Jersey, USA) into RIPA buffer (Solarbio, Beijing, China). The mixture was then heated at 100°C for 10 min. The lysates were separated by SDS-PAGE on a 7.5% polyacrylamide gel and transferred to a polyvinylidene fluoride membrane. The membrane was blocked in 5% bovine serum albumin plus Tween-20 buffered brine (TBST) and stored at room temperature for 1 h. The anti-DNAH3 antibody (rabbit polyclonal, self-made) was diluted 1:1,000 in TBST and incubated at 4°C overnight. Visualization was achieved using ECL luminescent solution (Affinibody LifeScience, Wuhan, HB, China), with β-actin used as a reference for protein levels.

### 2.7 Immunofluorescence staining

Immunofluorescence experiments were performed using human sperm that had been centrifuged and washed with sterile saline. The washed sperm was centrifuged again, and the pellet was resuspended in a small amount of saline. A 10- μl aliquot of the suspension was smeared onto a slide and air-dried at room temperature, followed by fixation in 4% PFA for 30 min. The slides were incubated at room temperature for 1 h in a blocking solution (5% bovine serum albumin and 0.1% Triton X-100 in 1× phosphate-buffered saline (PBS)). They were then incubated with primary antibodies overnight at 4°C. The primary antibodies used were rabbit polyclonal anti-DNAH3 (CUSABIO, Beijing, China), rabbit polyclonal anti-DNALI1 (Proteintech, Chicago, USA), and rabbit polyclonal anti-COXIV (Proteintech, Chicago, USA). Subsequently, the slides were washed three times with 1× PBS for 10 min each, followed by incubation with secondary antibodies (AlexaFluor 488/647 anti-rabbit, affinity; AlexaFluor 488/647 anti-mouse, affinity) and an appropriate amount of DAPI. The slides were then mounted with an anti-fluorescence quenching agent and observed for colocalization using a LEICA confocal microscope.

### 2.8 Intracytoplasmic sperm injection

**Figure 1:**
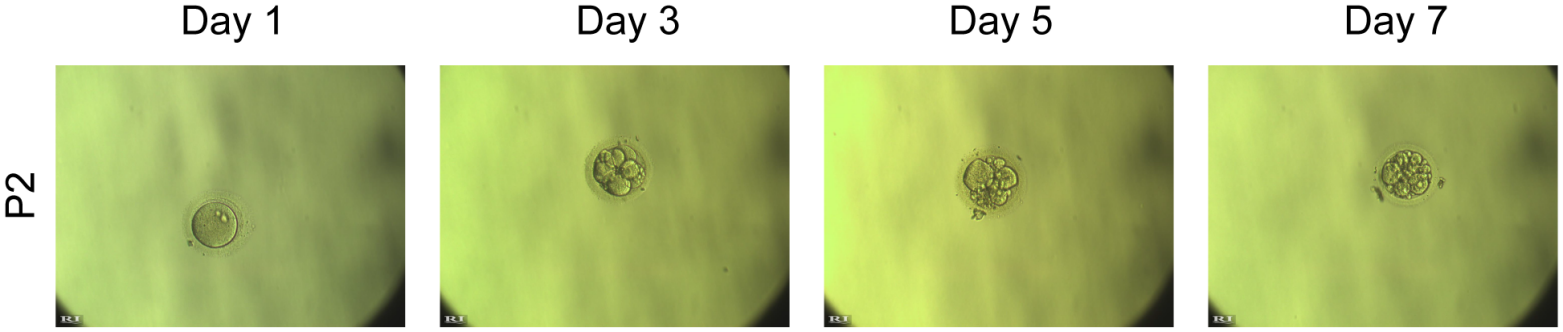
The embryo after ICSI injection from the patient 1.

To assess whether OAT-associated male infertility due to the DNAH3 mutation in this study could be treated with assisted reproductive technology, we performed intracytoplasmic sperm injection (ICSI) on the sperm from the second patient. The two-cell embryo and blastocyst rates in this proband were similar to those in the normal group (**Figure 1**). Patient (P2) underwent ICSI, resulting in the development of 12 embryos to the blastocyst stage. The patient’s wife received a single embryo transfer and eventually became pregnant.

### 2.9 Statistical analyses

Prism (version 9, GraphPad, Boston, MA, USA) and SPSS (Windows version 18.0, IBM, USA) were used to perform statistical analyses. All data are presented as means ± SEMs. Data from normal subjects and patients, including fertilization rate, blastocyst development rate, implantation rate, and clinical pregnancy rate, were analyzed using the *χ* ^2^ test. The means between the two groups were compared using an unpaired, parametric, two-sided Student’s *t*-test, with a *p*-value of less than 0.05 considered statistically significant.

## 3 Results

### 3.1 Identification of heterozygous DNAH3 mutations in men with severe OAT

**Figure 2:**
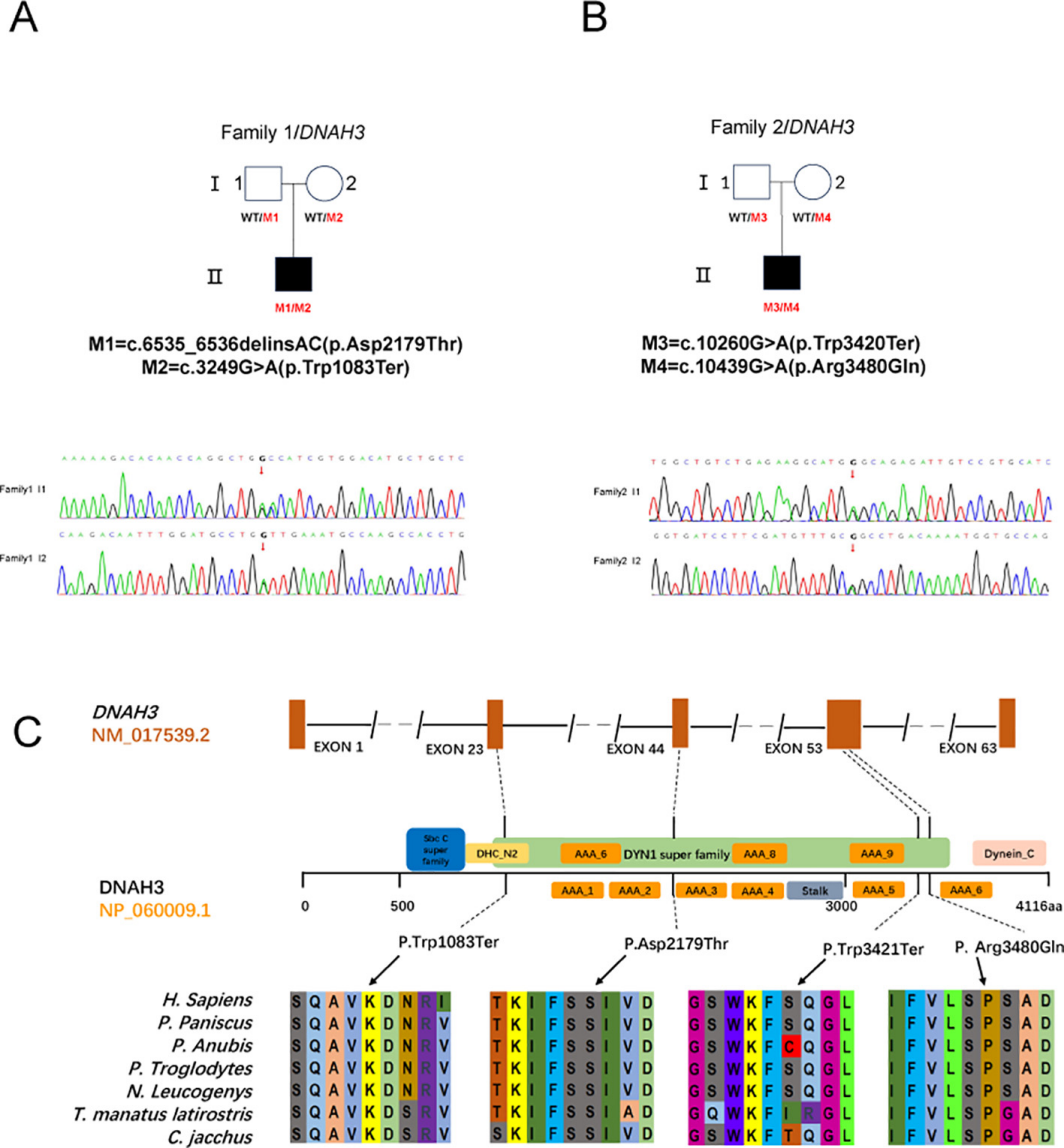
Pathogenic DNAH3 variants cause male infertility in humans.

In this study, after performing bioinformatic filtering analysis and Sanger sequencing comparison, we identified genes associated with OAT and found disease-causing double heterozygous mutation sites in DNAH3 in the male offspring of two nonconsanguineous families, which include four different heterozygous mutation sites. For the first patient (P1) in Family I, the biallelic mutation involved the deletion of two bases (GA) at positions 6535 and 6536, followed by the insertion of two bases (AC), resulting in the missense mutation (c.6535_6536 delinsAC; p.Asp2179Thr) and another stop codon premutation (c.3249G>A; p. Trp1083Ter) (**Figure 2A**). In family II, the patient (P2) harbored a DNAH3 heterozygous missense mutation (c. 10439G> A; p.Arg3480Gln) and a stop codon advance mutation (c.10260G > A; p.Trp3420Ter). The first mutation site involved a change from G to A at position 10260, leading to the creation of a premature stop codon that replaced the original Trp amino acid. The second mutation site involved a change from G to A at position 10439, resulting in the substitution of the original Arg amino acid with Gln (**Figure 2B**).

None of these DNAH3 mutations are present in the human population datasets of the 1,000 Genomes Project, the Exome Clustering Consortium, and the Genome Clustering Database. Notably, both DNAH3 mutations were also absent in racially matched control populations. For these two progenitors, low-frequency variants of other genes associated with male infertility were not found in the control population (**Table 1**). These new mutation sites are quite conserved across several different species (**Figure 2C**).

**Table 1:** Bi-allelic DNAH3 variants identified in OAT men.

	P1		P2	
cDNA alteration	c.6535_6536delinsA	c.3249G>A	c.10260G>A	c.10439G>A
Variant allele	Heterozygous	Heterozygous	Heterozygous	Heterozygous
Amino acid alteration	p.Asp2179Thr	p.Trp1083Ter	p.Trp3420Ter	p.Arg3480GIn
Variant type	Missense	Nonsense	Nonsense	Missense
Allele Frequency in Human Population				
gnomAD (v4.1.0)	0	0.0000031	0.0000068	0.0000465
1000 Genomes	0	0	0	0
Function Prediction				
PolyPhen-2	damaging	NA	NA	damaging
MutationTaster	damaging	damaging	damaging	damaging
SIFT	tolerated	NA	NA	damaging

NCBI reference sequence number of DNAH3 is NM_017539.2.

NA, not applicable.

### 3.2 DNAH3 is testicle-specific and closely related to spermatogenesis

**Figure 3:**
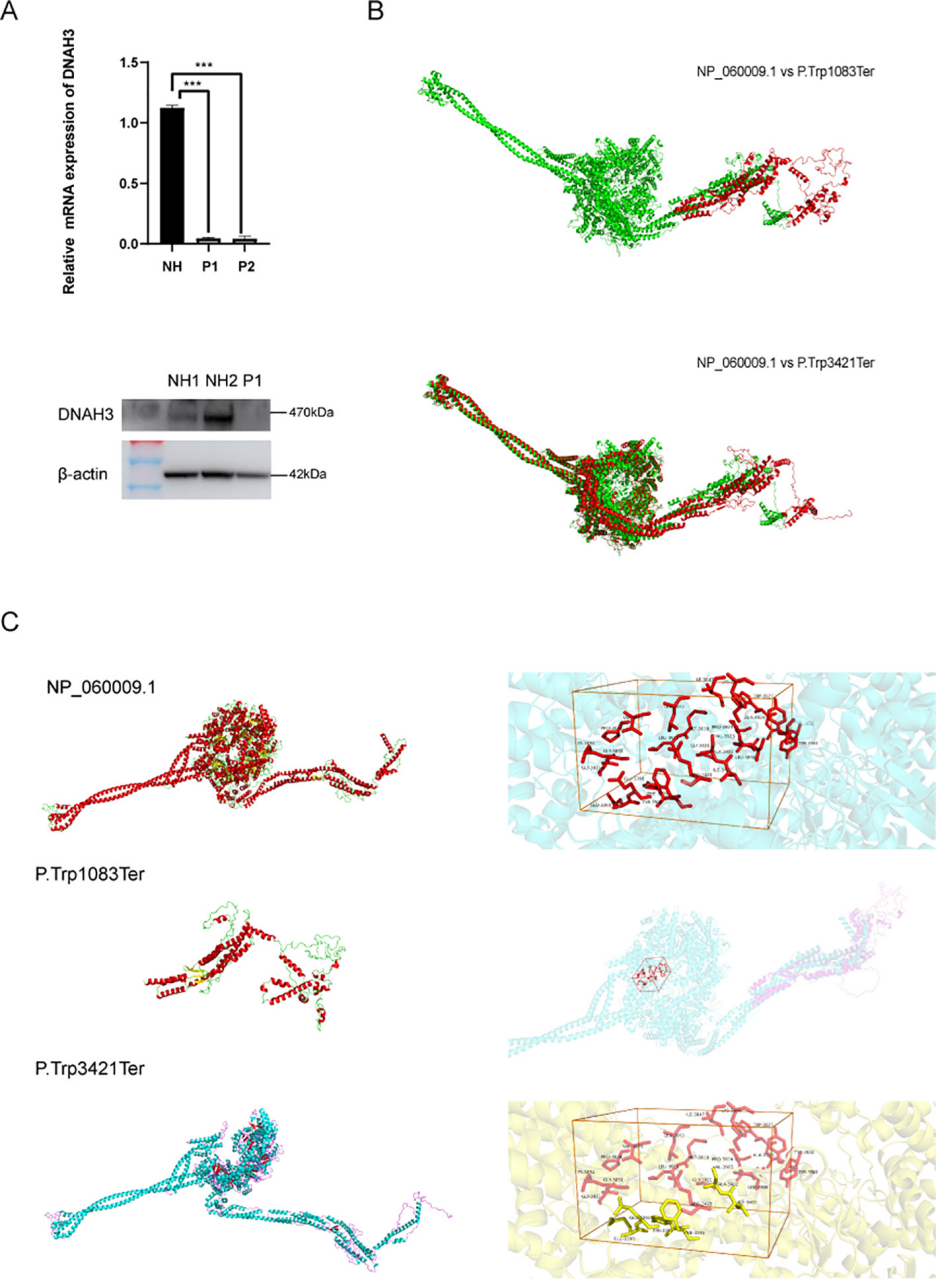
DNAH3 mutations display reduced Levels of DNAH3 and changed protein structure.

To further investigate the specific stages of spermatogenesis and differentiation at which dynamic changes in transcriptomic functional levels and associated gene expression occur, we examined the tissue-specific expression of DNAH3 in wild-type adult sexually mature mice. Our findings confirmed that DNAH3 is highly specific to the mouse testis, confirming the results of the NCBI literature search. Simultaneously, the testicles of wild-type C57 mice of different ages (in weeks) were analyzed by Western blotting (**Figure 3A**). In the mice, sperm began to form at 4 weeks of age, with DNAH3 mRNA and protein levels gradually increasing. The expression began to stabilize at 8 weeks of age and became established (**Figure 3B**).

### 3.3 OAT with severe MMAF in men with heterozygous DNAH3 mutations

**Figure 4:**
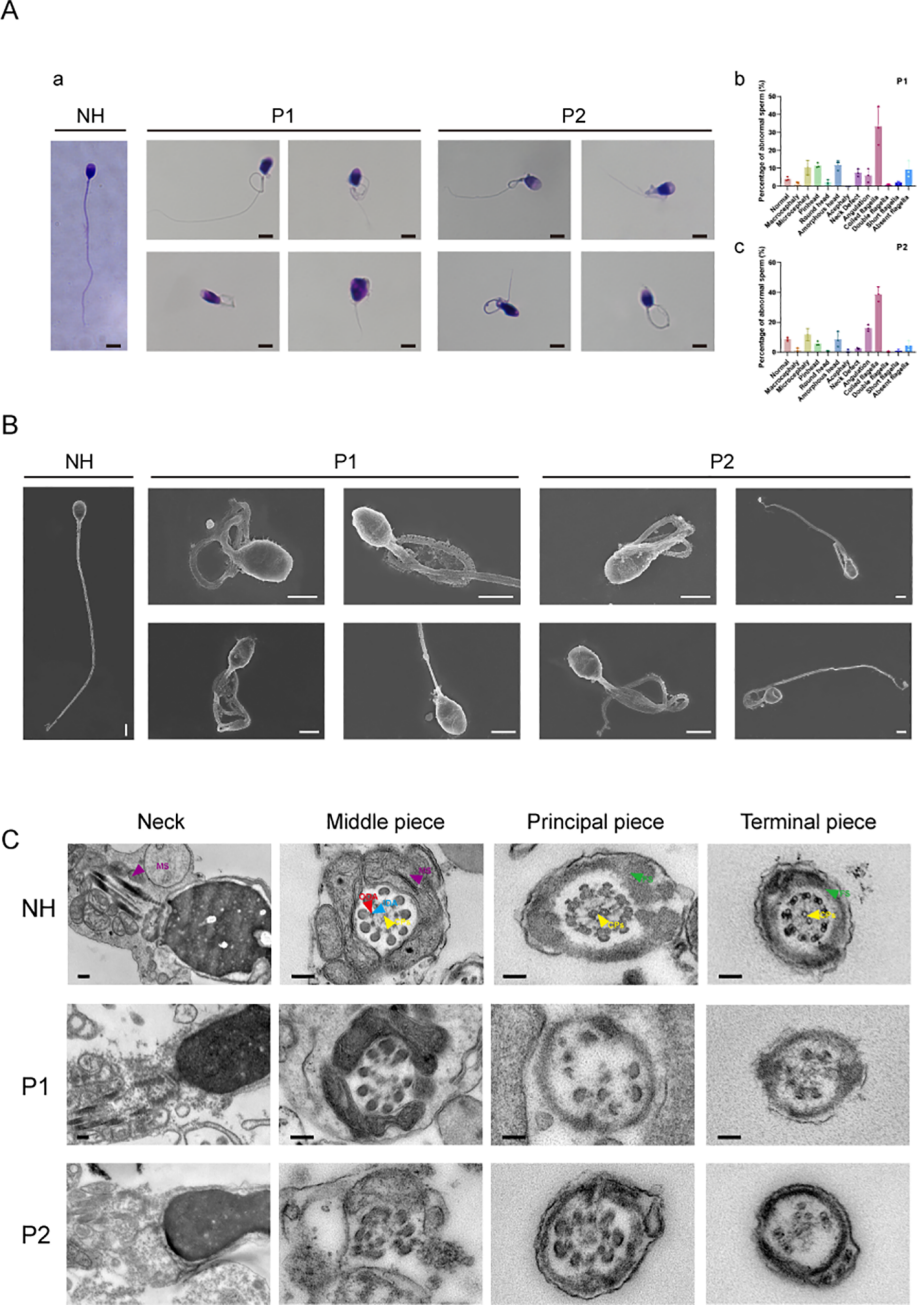
Verification of sperm abnormality in bi-allelic DNAH3 mutation men with severe OAT.

We conducted a detailed analysis of the phenotypes of sperm diseases following biallelic heterozygous mutations in two cases. Semen was collected from patients after an abstinence period of more than 3–5 days, liquefied at 37°C for 30 min, and then evaluated. Through the collection and examination of clinical semen and sperm analyses, we found that the corresponding clinical indicators for the two patients were greatly reduced compared to the standard parameters, including semen volume, sperm concentration, sperm motility, progressive sperm motility, and sperm morphology during routine clinical diagnosis (**Table 2**). We counted 300 sperm using Papanicolaou staining, analyzing more than 100 sperm at a time. Compared with normal patients, the two men with DNAH3 mutations showed a variety of abnormalities in sperm morphology, including sperm neck defects, short flagella, and deletions (**Figure 4A**). These abnormalities were more severe than those reported in patients with DNAH3 mutations in previously published work (27). The first patient had typical malformed sperm phenotypes, including head malformations and neck defects. The second patient mainly presented with schizocauda and curvilinear spermia. It is worth noting that tail malformations accounted for more than 60% of the total sperm in patients with DNAH3 mutations.

**Table 2:** Semen characteristics of men carrying bi-allelic DNAH3 variants.

Semen parameters*	Human subject		Normal value of WHO criteria (The sixth edition)
	**P1**	**P2**	
Semen volume (mL)	4.2	4	1.4-5.6
Sperm concentration(106/mL)	0.2*	0.7*	≥15.0
Total sperm number (106/ejaculate)	1.4*	2.8*	≥39
Sperm motility (%)	83.3	57.1	≥40
Progressive motility (%)	75	57.1	≥32
Normal sperm morphology(%)	4*	8	≥4
Leukocyte concentration(106/mL)	1.2	1	<1.0

The normal values of semen parameters were according to the WHO (2021) manual criteria. Semen analyses were performed twice for each subject.

*Abnormal values.

The sperm from normal individuals who underwent semen examination were used as controls. SEM and TEM analyses confirmed severe flagellar abnormalities (**Figures 4B, C**). Compared to the normal samples, the sperm section samples from the two patients have different degrees of deletion and disintegration in different structures (which have been labeled in the figure), especially in the 9 + 2 structure and the dynamin junction. In particular, the structure of the mitochondrial sheath of the flagella was also damaged in the first patient, corresponding with the phenotype observed in Papanicolaou staining and SEM. In conclusion, the structure and function of motility proteins in the sperm of the two patients with a biallelic heterozygous mutation in DNAH3 had varying degrees of change, affecting sperm development and structure.

### 3.4 DNAH3 mutations cause changes in RNA transcription levels and protein translation functions

**Figure 5:**
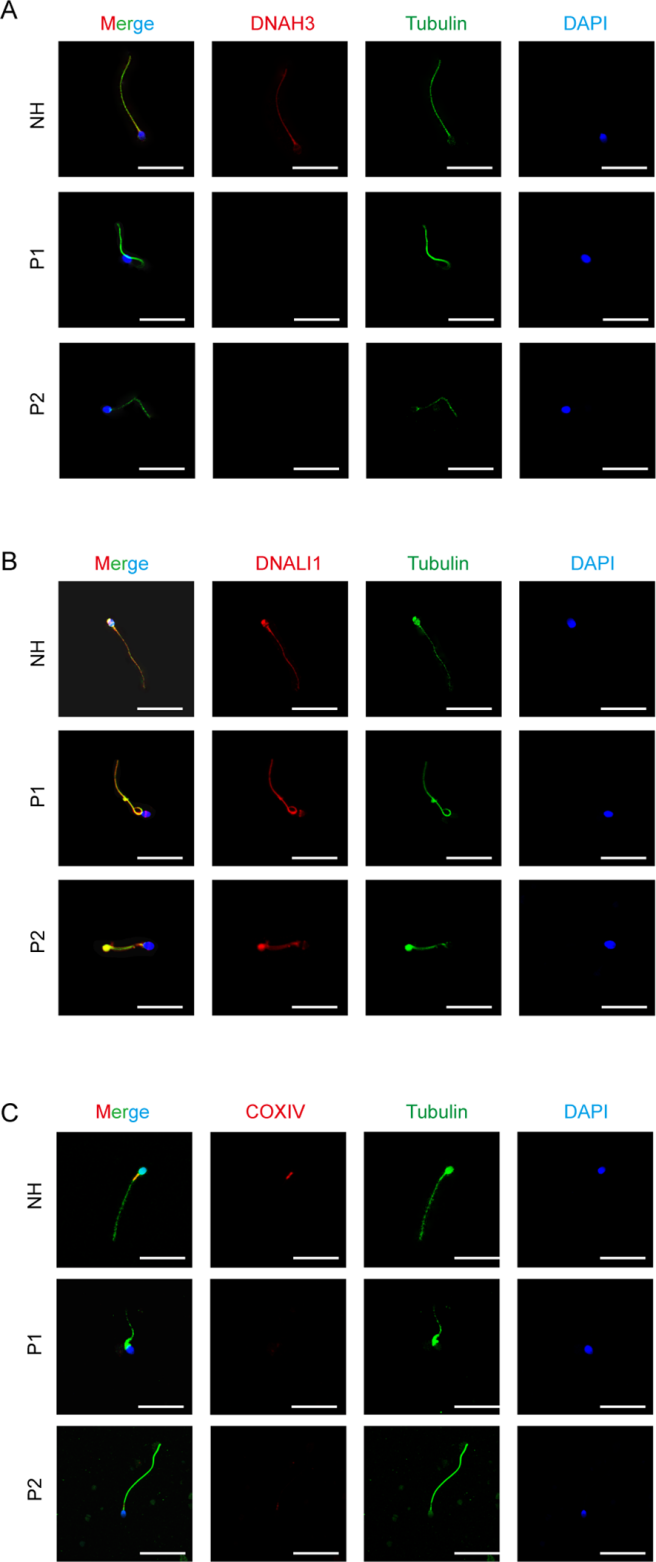
Validation of DNAH3 mutations resulting in altered levels of related proteins in sperm.

RT-qPCR and Western blotting experiments were conducted to verify the *in vitro* effect of the biallelic heterozygous mutation in the patient’s semen. As shown in **Figure 5**, the mRNA and protein expression levels of DNAH3 in the patient were significantly reduced (**Figure 5A**). In related published literature (27), it is mentioned that the protein levels in patient sperm are relatively lower than in normal sperm. The two biallelic heterozygous mutations we refer to here involve base mutations that advance the terminator codon. We used SWISS-MODEL (https://swissmodel.expasy.org/) to construct the three-dimensional structure of the wild-type DNAH3 protein (NP_060009.1) and the two terminator mutation variants for online prediction. Using homology modeling and combining QMEAN and GMQE value ranges, we selected the model with the highest similarity value and sequence homology for structural prediction. It can be seen that the two protein variants of DNAH3 with advanced terminator mutations exhibit significant changes in amino acid secondary and tertiary structures (**Figure 5B**). In addition, PyMOL was used to splice the wild-type DNAH3 protein variant and the advances terminator protein variant.

Subsequently, we predicted the changed protein active pockets using Protein Plus and selected those with the highest predictive scores for visualization. After comparing the amino acid residues in these pockets with the protein structures of M2 and M3, we found that the M3 protein was missing some amino acid residues that comprise the active pockets, while the M2 protein completely lacks this active pocket (**Figure 5C**).

It is obvious that the mRNA expression level, protein expression, and structure of DNAH3 change after mutation, affect ing the male clinical reproductive phenotype.

### 3.5 Validation of DNAH3 mutations resulting in altered expression of related proteins in sperm

**Figure 6:**
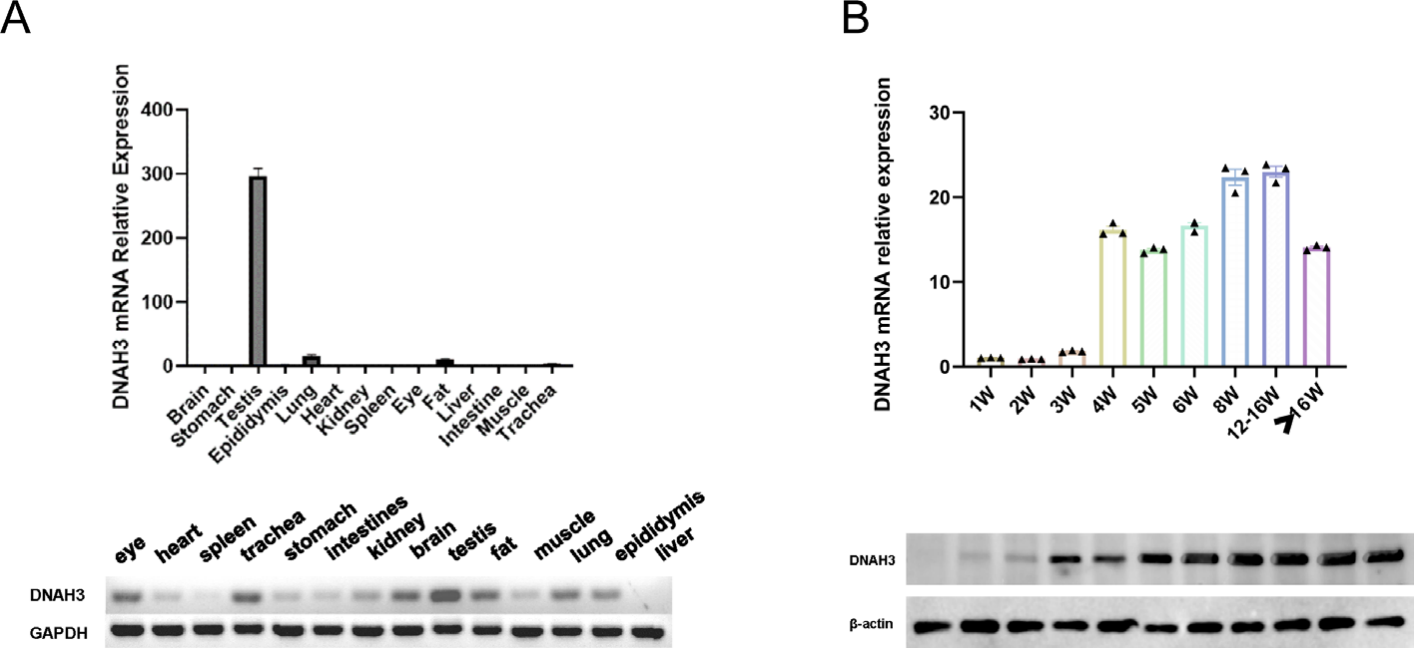
DNAH3 is testicle-specific and closely related to spermatogenesis.

We performed immunofluorescence staining on sperm smears of healthy individuals and two patients to assess localization and expression changes of DNAH3, DNALI1, and COXIV. In fertile controls, DNAH3 immunostaining was concentrated in the sperm neck and flagella, whereas it was absent in the sperm of patients with DNAH3 mutations (**Figure 6A**). In other IDAs, we selected DNALI1 for staining and observed that, in healthy sperm, DNALI1 was expressed throughout the sperm. In contrast, the staining of DNALI1 in the sperm of patients with DNAH3 mutations lacked integrity (**Figure 6B**). Subsequently, we stained COXIV, an important component of the mitochondrial oxidative phosphorylation chain that plays a role in cellular energy metabolism. Given the distribution of mitochondria in sperm, COXIV is expressed in the mitochondrial sheath, particularly in the neck region of the sperm (36). In the two patients, COXIV staining was significantly lost or weakened (**Figure 6C**). We verified changes in the protein expression of some genes associated with DNAH3 in sperm samples from normal and healthy individuals and examined how DNAH3 mutations lead to altered sperm phenotypes through changes in protein function.

### 3.6 Patient with the new DNAH3 mutation obtained positive intracytoplasmic sperm injection result

The successful clinical ICSI outcomes came from the second patient (P2) in this study, which eventually led to a positive pregnancy. For proband P2, his 30-year-old wife underwent a single ICSI cycle, retrieving 14 mature oocytes and 12 high-quality blastocysts. After embryo transfer, a successful live birth was achieved. The first patient (P1) did not receive ICSI treatment and lacked ICSI data. Despite this, we recommend ICSI treatment for patients with DNAH3 mutations.

## 4 Discussion

This study provides an in-depth exploration of the complex relationship between biallelic mutations in the *DNAH3* gene and the phenotype of OAT, a common form of male infertility. The growing body of evidence implicates the dynein family of proteins, particularly DNAH3, which plays a vital role in the intricate processes of spermatogenesis and sperm motility.

We found two patients with novel biallelic mutations in DNAH3 among 128 unrelated Chinese men. Both probands exhibited a heterozygous mutation that advanced the stop codon, affecting the mRNA and protein expression levels of DNAH3. The mutations identified in this study may lead to the truncation of proteins that impair the motility function of dynein, resulting in the characteristic OAT phenotype observed in the patients. DNAH3, a member of the axonemal dynein family, plays an indispensable role in the structure and function of sperm flagella. Therefore, we believe that the more severe phenotype with OAT was observed in this study's patients, along with the advancement of the DNAH3 stop codon, due to changes in both protein expression and protein structure. These changes affect the abnormal function of DNAH3 in spermatogenesis and component composition. Future studies should focus on specific mechanism s, including conducting robust sperm proteomic analyses to enhance the understanding and impact of sperm proteomic data (37).

The identification of premature stop codons in DNAH3 suggests that these mutations lead to a loss of function, manifesting as severe OAT. The phenotypes observed in our patients, including reduced sperm motility and multiple morphological abnormalities of the sperm flagella (MMAF), are consistent with the known functions of DNAH3 in flagellar assembly and motility. This study reinforces the notion that DNAH3 is not just a candidate gene but a significant contributor to the pathogenesis of OAT.

Immunofluorescence staining and subsequent analysis of protein expression in sperm samples from our patients revealed significant changes in the localization and expression levels of proteins associated with DNAH3. The absence or reduction of DNAH3 immunostaining in the sperm of patients with mutations confirms the direct impact of these mutations on protein expression. In addition, the observed changes in the expression of other dynein arms and mitochondrial sheath proteins highlight the interconnected nature of flagellar assembly and the potential pleiotropic effects of DNAH3 mutations.

Fortunately, P2 patients were able to reproduce using ICSI treatment (**Figure 1**). The clinical phenotype of our patient is characterized by severe OAT, highlighting the potential application of assisted reproductive technologies, such as ICSI, in the treatment of male infertility associated with DNAH3 mutations. We will also continue to observe babies born through *in vitro* fertilization (IVF) and keep long-term health records. The patient's sperm with normal head shape can be found even if the flagella are not developed or are stunted. Most mutants are sterile and occasionally semisterile. For completeness, the influence of sex chromosomes on sperm phenotypes is also included. Functionally, the genes involved can be classified as regulators of spermatogenesis. When using human-assisted reproductive technology to aid sperm competition, it is possible to assume that short-term single-phase fitness (i.e., motility) is combined with appropriate nuclear maturation during development, including the protection of genetic integrity of multigenerational fitness (38). Our findings suggest that despite the severe phenotype, ICSI may provide a viable reproductive option for individuals affected by early termination mutations due to DNAH3 termination codons.

The DNAH3 gene will serve as a new indicator of pathogenic genes that have not received much attention in the field of male infertility. Further studies are needed to explore the therapeutic potential of modulating the expression or function of DNAH3 in the treatment of OAT. Additionally, the identification of other genes in the dynein family or related pathways that may contribute to OAT could provide new insights and therapeutic targets.

In summary, our study provides compelling evidence for the role of DNAH3 mutations in the etiology of OAT. The identification of these mutations and their phenotypic consequences not only enhances our understanding of the molecular basis of male infertility but also has significant implications for the clinical management of affected individuals. The potential of gene therapy and other targeted interventions to address the underlying genetic defects warrants further investigation.

## Data Availability

The original contributions presented in the study are included in the article/**Supplementary Material**, further inquiries can be directed to the corresponding authors.
