# Supplementary material for: Novel bi-allelic DNAH3 variants cause oligoasthenoteratozoospermia
Source: Front Endocrinol (Lausanne). 2024 Oct 28;15:1462509. doi: 10.3389/fendo.2024.1462509 (PMC11586517; doi:10.3389/fendo.2024.1462509)
Supplement: Supplementary file 1 [file Table1.docx]

| **Table S1 Real-time RT-PCR primer sequences** | | | | |
| --- | --- | --- | --- | --- |
| **Genes** | **Species** | **Forward sequence(5’_3’)** | **Reverse sequence(5’_3’)** | **Product sizes (bp)** |
| *Dnah3* | Mouse | TTTGGCACCCCTGTCTTGTT | GGAGACAGACTTTCACGGCA | 214 |
| *DNAH3* | Human | CATGTCCAAGCTGGATCGACGA | CCATACACCTCTTTGGCTGGCA | 108 |
| *Gapdh* | Mouse | CATCACTGCCACCCAGAAGACTG | ATGCCAGTGAGCTTCCCGTTCAG | 153 |
| *GAPDH* | Human | GTCTCCTCTGACTTCAACAGCG | ACCACCCTGTTGCTGTAGCCAA | 131 |
