# Supplementary figures and images for: Novel bi-allelic DNAH3 variants cause oligoasthenoteratozoospermia
Source: Front Endocrinol (Lausanne). 2024 Oct 28;15:1462509. doi: 10.3389/fendo.2024.1462509 (PMC11586517; doi:10.3389/fendo.2024.1462509)

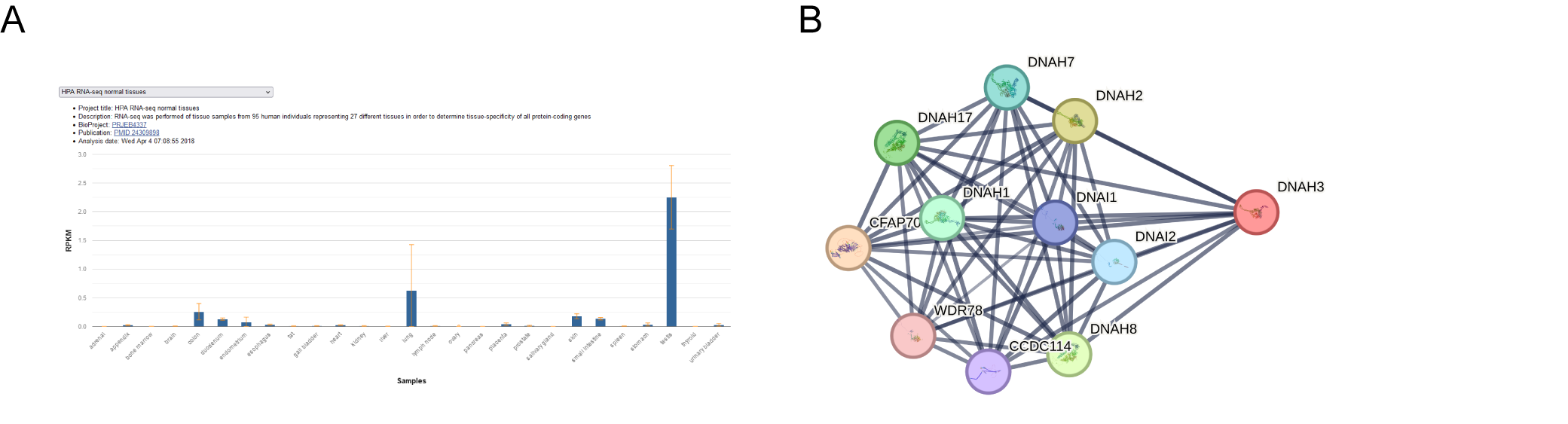

Supplement: Supplementary file 2 [file Image1.tif]
